# Editorial: Vaginal dysbiosis and biofilms, volume II

**DOI:** 10.3389/fcimb.2025.1588434

**Published:** 2025-03-19

**Authors:** António Machado, Claudio Foschi, Antonella Marangoni

**Affiliations:** ^1^ Universidade dos Açores, Faculdade de Ciências e Tecnologia, Departamento de Biologia, Centro de Biotecnologia dos Açores (CBA), Ponta Delgada, Portugal; ^2^ Universidad San Francisco de Quito USFQ, Colegio de Ciencias Biológicas y Ambientales COCIBA, Instituto de Microbiología, Laboratorio de Bacteriología, Quito, Ecuador; ^3^ Microbiology, Department of Experimental, Diagnostic and Specialty Medicine, University of Bologna, Bologna, Italy; ^4^ Microbiology Unit, IRCCS Azienda Ospedaliero-Universitaria of Bologna, Bologna, Italy

**Keywords:** biofilms, vaginal dysbiosis, reproductive health, vaginal microbiota, accurate diagnostics, antimicrobial resistance, novel treatments

The vaginal microbiota is a dynamic ecosystem essential for reproductive health, predominantly maintained by peculiar *Lactobacillus* species. These bacteria produce lactic acid, creating an acidic environment that inhibits pathogen colonization (Machado et al., 2013; Rodríguez-Arias et al., 2022; Morselli et al., 2024). However, disruptions in this balance lead to vaginal dysbiosis, associated with bacterial vaginosis (BV), vulvovaginal candidiasis (VVC), and aerobic vaginitis (AV) (Salinas et al., 2018). Biofilms, structured microbial communities encased in an extracellular matrix, play a crucial role in vaginal dysbiosis (see **Figure 1**), such as BV, by enhancing pathogen persistence and resistance to antimicrobials (Muñoz-Barreno et al., 2021). Vaginal dysbiosis is associated with an increased risk of acquiring sexually transmitted infections, such as human immunodeficiency virus (HIV), Herpes simplex type 2, and *Chlamydia trachomatis*, as well as an increased frequency of reproductive complications (Parolin et al., 2015; Ceccarani et al., 2019; De Gregorio et al., 2020).

**Figure 1:**
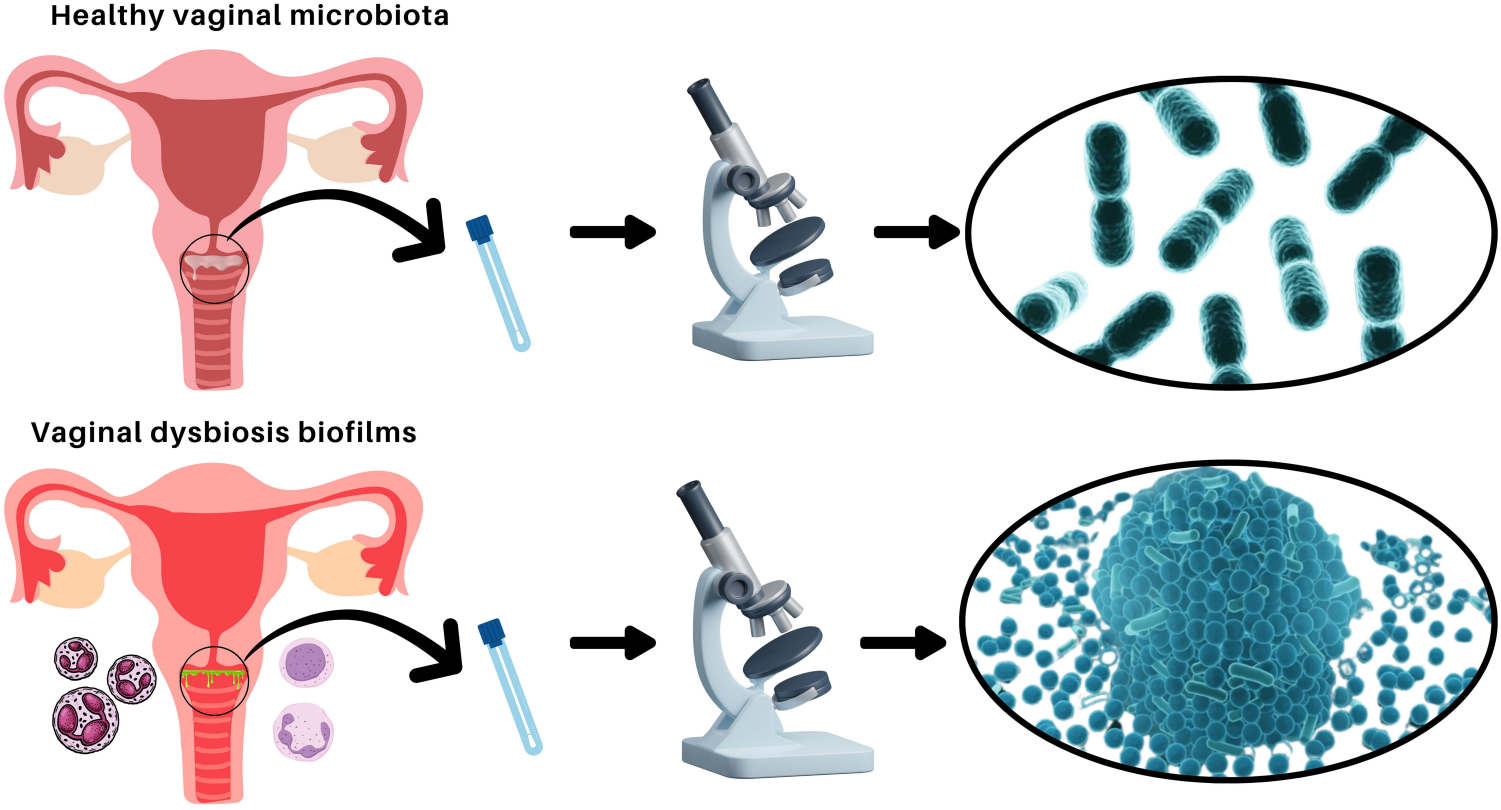
Comparison of healthy vaginal microbiota and vaginal dysbiosis with biofilm formation. Illustration depicting the differences between a balanced vaginal microbiota dominated by *Lactobacillus* species (top) and vaginal dysbiosis characterized by pathogenic biofilm formation (bottom). In a healthy state, the vaginal microbiota consists of beneficial bacteria that maintain an acidic pH and protect against infections. In dysbiosis, biofilms formed by opportunistic pathogens contribute to persistent infections and increased resistance to treatments.

This second volume of the Research Topic expands on the complex interactions between microbial communities, host responses, and biofilms (Machado et al., 2022). The articles included explore novel diagnostic methods, targeted therapies, and probiotic interventions aimed at restoring microbial balance. Given the challenges posed by biofilm-associated infections, understanding their molecular mechanisms is essential for developing more effective treatments. By advancing research on vaginal dysbiosis and biofilms, this Research Topic seeks to improve diagnostic accuracy and therapeutic strategies (Henriques et al., 2012; Machado et al., 2015), ultimately enhancing women’s reproductive health and preventing long-term complications (De Gregorio et al., 2020).

Zierden et al. explored the influence of the vaginal microbiome on cervicovaginal mucus (CVM) barrier properties during pregnancy. The authors collected CVM samples from pregnant participants and used multiple particle tracking (MPT) and *16S* rRNA sequencing to analyze barrier function and microbiome composition. Their findings revealed that *Lactobacillus crispatus*-dominated microbiota was associated with stronger CVM barrier function, while polymicrobial communities increased permeability. This suggests that microbial composition plays a crucial role in preventing bacterial ascension, with potential implications for preterm birth risk. Understanding these interactions could aid in the development of vaginally delivered therapeutics to support reproductive health during pregnancy. Building on the role of beneficial microbes in vaginal health, Takano et al. evaluated how *Lactobacillus* species inhibit *Candida albicans* growth, biofilm formation, and epithelial adhesion. By analyzing 27 *Lactobacillus* strains, the study identified lactate as a key factor in suppressing *C. albicans* biofilms and hyphal transition, while some strains also significantly reduced fungal adhesion to epithelial cells. These findings highlight the potential of *Lactobacillus*-derived metabolites as alternative antifungal strategies, offering a promising avenue for preventing vulvovaginal candidiasis.

Given the importance of accurately characterizing vaginal microbiota for clinical and research applications, Short et al. compared two sampling techniques in pregnant women living with HIV-1: menstrual cups and high vaginal swabs. The study found no significant differences in bacterial load, composition, or diversity between methods, validating both for microbiota analysis. Menstrual cups, however, collected a larger sample volume, making them a practical alternative for self-sampling and expanded laboratory analysis. This research underscores the need for adaptable and efficient methodologies in vaginal microbiota studies, particularly for populations with increased reproductive health risks.

Furthermore, Mao et al. studied the association between vaginal and cervical microbiome dysbiosis and uterine fibroids. By analyzing microbial profiles from 29 women with uterine fibroids and 38 healthy controls, the study found no significant difference in overall microbial diversity. However, alpha diversity was negatively correlated with the number of fibroids, and an increased abundance of *Firmicutes* was observed in fibroid patients. Certain bacterial genera were significantly enriched or depleted, indicating microbial alterations linked to fibroid presence. The findings suggest that microbiome disruptions may contribute to fibroid pathogenesis, offering new insights for potential preventive and therapeutic strategies. The impact of vaginal microbiota on systemic health is further exemplified in the case study by Liu et al., which reported a rare instance of *Fannyhessea vaginae* bacteremia in a pregnant woman with bacterial vaginosis. Blood cultures confirmed *F. vaginae* as the causative agent, and the patient responded well to cefoperazone/sulbactam treatment. This study highlights the clinical significance of anaerobic vaginal pathogens, particularly in pregnant women, where microbial imbalances can lead to severe complications. A review of previous cases reinforced *F. vaginae*’s association with bacterial vaginosis, preterm birth, and systemic infections, emphasizing the need for heightened awareness of its pathogenic potential.

Beyond microbial composition, hormonal regulation plays a key role in shaping the vaginal microbiota, as explored by Rahman et al. Using a mouse model, they demonstrated that estrogen significantly influences *Lactobacillus* and *Gardnerella vaginalis* colonization. Mice treated with 17β-estradiol exhibited increased glycogen levels, which supported *Lactobacillus* colonization, whereas progesterone alone failed to restore microbial balance. These findings suggest that sex hormones modulate vaginal microbiota stability, offering potential therapeutic avenues for managing dysbiosis through hormonal interventions.

Zhang et al. reviewed the relationship between vaginal microbiota, human papillomavirus (HPV) infection, and cervical cancer. They highlighted how vaginal dysbiosis, characterized by reduced *Lactobacillus* abundance and increased microbial diversity, contributes to HPV persistence and cervical lesion progression. This review underscores the complex interactions between microbial communities and HPV, providing a basis for future research into personalized diagnostic and treatment strategies. Meanwhile, Chen et al. analyzed the pathogenic function of sialidases in BV. Sialidases produced by *Gardnerella vaginalis* and other anaerobes degrade the protective mucus layer of the vaginal epithelium, facilitating bacterial adhesion, biofilm formation, and immune evasion. The study reviewed sialidase-based diagnostic tools and therapeutic potential, suggesting that sialidase inhibitors could be promising pharmacological targets for BV treatment. On the other hand, Cao et al. evaluated the synergistic effects of Kangbainian (KBN) lotion and miconazole nitrate (MN) against drug-resistant *Candida albicans* biofilms. *In vitro* assays revealed that the combination of KBN and MN disrupted biofilm integrity, reduced fungal viability, and downregulated key biofilm-associated genes. This study highlights the growing need for novel antifungal strategies to combat drug-resistant biofilms in vaginal infections.

Finally, Himschoot et al. investigated the prevalence and clinical correlations of *Gardnerella* species, *Fannyhessea vaginae*, *Lactobacillus crispatus*, and *L. iners* in pregnant women in the Democratic Republic of the Congo. By analyzing samples from 331 pregnant women, they found that *L. iners* was the most prevalent species, while *G. vaginalis* was the most common *Gardnerella* species. Notably, *F. vaginae* was identified as the best molecular marker for bacterial vaginosis (BV), with a high diagnostic performance. The study also highlighted associations between microbial species and BV-related symptoms, as well as potential links between *L. iners* and preterm birth. Last, but not least, in the eleventh article, Zheng et al. assessed the role of reproductive tract microbiota in gynecological diseases. The review explored microbial alterations in conditions such as endometrial polyps, uterine fibroids, endometriosis, adenomyosis, and endometrial cancer, highlighting the potential of microbiota as both diagnostic markers and therapeutic targets. The review suggested that certain bacterial species, such as *F. vaginae*, may contribute to disease progression, while microbiota-targeted interventions, including probiotics and microbiome transplants, offer promising treatment avenues. The integration of microbiome research with other omics sciences like transcriptomics and proteomics could further refine diagnostic and therapeutic strategies.

## References

[B1] Ceccarani C. Foschi C. Parolin C. D’Antuono A. Gaspari V. Consolandi C.. (2019). Diversity of vaginal microbiome and metabolome during genital infections. Sci. Rep. 9, 1–12. doi: 10.1038/s41598-019-50410-x 31575935 PMC6773718

[B2] De Gregorio P. R. Parolin C. Abruzzo A. Luppi B. Protti M. Mercolini L.. (2020). Biosurfactant from vaginal Lactobacillus crispatus BC1 as a promising agent to interfere with Candida adhesion. Microb. Cell Fact 19, 1–16. doi: 10.1186/s12934-020-01390-5 32552788 PMC7302142

[B3] Henriques A. Cereija T. Machado A. Cerca N. (2012). In silico vs *in vitro* analysis of primer specificity for the detection of Gardnerella vaginalis, Atopobium vaginae and Lactobacillus spp. BMC Res. Notes 5, 637. doi: 10.1186/1756-0500-5-637 23153093 PMC3522034

[B4] Machado A. Castro J. Cereija T. Almeida C. Cerca N. (2015). Diagnosis of bacterial vaginosis by a new multiplex peptide nucleic acid fluorescence in *situ* hybridization method. PeerJ 3, e780. Available at: https://peerj.com/articles/780/ (Accessed March 01, 2025). 25737820 10.7717/peerj.780 PMC4338769

[B5] Machado A. Foschi C. Marangoni A. (2022). Editorial: Vaginal dysbiosis and biofilms. Front. Cell Infect. Microbiol. 12. doi: 10.3389/fcimb.2022.976057 PMC9396345 36017371

[B6] Machado A. Salgueiro D. Harwich M. Jefferson K. K. Cerca N. (2013). Quantitative analysis of initial adhesion of bacterial vaginosis-associated anaerobes to ME-180 cells. Anaerobe 23, 1–4. doi: 10.1016/j.anaerobe.2013.07.007 23916636

[B7] Morselli S. Ceccarani C. Djusse M. E. Laghi L. Camboni T. Consolandi C.. (2024). Anti-chlamydial activity of vaginal fluids: new evidence from an *in vitro* model. Front. Cell Infect. Microbiol. 14. doi: 10.3389/fcimb.2024.1403782 PMC11193362 38912205

[B8] Muñoz-Barreno A. Cabezas-Mera F. Tejera E. Machado A. (2021). Comparative effectiveness of treatments for bacterial vaginosis: A network meta-analysis. Antibiotics 10, 1–16. doi: 10.3390/antibiotics10080978 PMC8388924 34439028

[B9] Parolin C. Marangoni A. Laghi L. Foschi C. Palomino R.A. Ñ. Calonghi N.. (2015). Isolation of vaginal lactobacilli and characterization of anti-candida activity. PloS One 10, 1–17. doi: 10.1371/journal.pone.0131220 PMC4476673 26098675

[B10] Rodríguez-Arias R. J. Guachi-Álvarez B. O. Montalvo-Vivero D. E. Machado A. (2022). Lactobacilli displacement and Candida albicans inhibition on initial adhesion assays: a probiotic analysis. BMC Res. Notes 15, 1–7. doi: 10.1186/s13104-022-06114-z 35799214 PMC9264498

[B11] Salinas A. M. Osorio V. G. Endara P. F. Salazar E. R. Vasco G. P. Vivero S. G.. (2018). Bacterial identification of the vaginal microbiota in Ecuadorian pregnant teenagers: an exploratory analysis. PeerJ 6, e4317. doi: 10.7717/peerj.4317 29492333 PMC5826987

